# Optimizing bronchodilation in the prevention of COPD exacerbations

**DOI:** 10.1186/s12931-017-0601-2

**Published:** 2017-06-20

**Authors:** Marc Miravitlles, Antonio Anzueto, José R. Jardim

**Affiliations:** 1 Pneumology Department, Hospital Universitari Vall d’Hebron. CIBER de Enfermedades Respiratorias (CIBERES), Barcelona, Spain; 2 University of Texas Health Science Center, and South Texas Veterans Health Care System, San Antonio, TX USA; 3 Respiratory Division, Escola Paulista de Medicina / Federal University de São Paulo, São Paulo, Brazil

**Keywords:** Dual bronchodilation, ICS, LABA, LAMA, Treatment guidelines, Triple therapy

## Abstract

The natural disease course of chronic obstructive pulmonary disease (COPD) is often punctuated by exacerbations: acute events of symptom worsening associated with significant morbidity and healthcare resource utilization; reduced quality of life; and increased risk of hospitalization and death. The Global Initiative for Chronic Obstructive Lung Disease (GOLD) recommend that patients at risk of exacerbations (GOLD Groups C and D) receive a long-acting muscarinic antagonist (LAMA) or a long-acting β_2_-agonist (LABA)/LAMA combination, respectively, as preferred initial treatments. The latter recommendation is based on recent trial evidence demonstrating the superior efficacy of a fixed-dose LABA/LAMA over an inhaled corticosteroid (ICS)/LABA in exacerbation prevention. ICS in combination with a LABA is also indicated for prevention of exacerbations, but the use of ICS is associated with an increased risk of adverse events such as pneumonia, and offers limited benefits beyond those provided by LABA or LAMA monotherapy. In this review, we examine evidence from a number of pivotal studies of LABAs and LAMAs, administered as monotherapy or as part of dual or triple combination therapy, with a specific focus on their effect on exacerbations. We also discuss a new proposed treatment paradigm for the management of COPD that takes into account this recent evidence and adopts a more cautious approach to the use of ICS. In alignment with GOLD 2017, we suggest that ICS should be reserved for patients with concomitant asthma or in whom exacerbations persist despite treatment with LABA/LAMA.

## Background

The natural trajectory of chronic obstructive pulmonary disease (COPD) is punctuated by exacerbations, defined as an acute worsening of symptoms that results in additional therapy [1, 2]. In many cases, exacerbations are triggered by respiratory tract infections (predominantly viral, but also bacterial) and environmental factors such as air pollution, yet in approximately one third of cases, the cause remains unknown [3].

COPD exacerbations have a marked negative effect on both the patient and underlying disease processes, and can result in hospitalization and readmission, an increased risk of death [4] and a significant reduction in health status [5, 6]. Exacerbations are also associated with long-term decline in lung function and a high socioeconomic cost [7–10]. Thus, optimizing the prevention and management of COPD exacerbations is an important clinical issue.

A key step towards meeting this goal is to identify patients at greatest risk of exacerbation. The ‘frequent exacerbator’ phenotype (≥2 exacerbations/year) describes patients who are particularly susceptible to exacerbations, in contrast to infrequent exacerbators [11, 12]. The exacerbator phenotype, which remains relatively stable over time [12], has a complex pathophysiology and is prevalent across all disease severities, but is more common in patients with worse lung function [12].

The Global Initiative for Chronic Obstructive Lung Disease (GOLD) provides treatment recommendations for patients who are at high risk of exacerbation: a long-acting muscarinic antagonist (LAMA) or a long-acting β_2_-agonist (LABA)/LAMA combination are recommended as primary choice treatment for these patients. Although the GOLD strategy document was developed with an international audience in mind, some countries and regions (e.g. Spain, UK, South America, and Canada) follow guidance outlined in their own recommendations as well [13–16].

Inhaled corticosteroids (ICS) are recommended for patients at high risk of exacerbation with concomitant asthma, or who experience further exacerbations following initial bronchodilator treatment [2]. However, there is widespread evidence of inappropriate use of ICS in patients at low risk of exacerbation [17, 18]. Considering the known risks associated with long-term ICS use, only patients carefully considered as suitable candidates should receive treatment with the appropriate ICS and associated dose, such that treatment benefit will outweigh any potential risk [19].

Strong evidence supports the favorable efficacy and safety profile of dual bronchodilators. Compared with bronchodilator monotherapy and ICS/LABA, LABA/LAMAs improve lung function [20–29] and reduce exacerbation risk [23, 24, 28, 29]. The safety profile of LABA/LAMA combinations is generally similar to that of placebo or individual monocomponents [22, 30–33]. However, LABA/LAMA use is still relatively limited and further experience with these agents is needed [34].

In this review, we will examine the efficacy of various COPD treatments in terms of exacerbation prevention, with particular focus on bronchodilators, and discuss a new proposed treatment paradigm for reducing exacerbation risk in patients with COPD.

## Efficacy Of Bronchodilators In Prevention Of Exacerbations

### Non-pharmacological intervention

Smoking cessation is the most effective initial strategy for reducing COPD disease progression, particularly exacerbation risk [2, 35]. Smoking cessation significantly reduces the progressive decline in lung function [36], and substantially lowers the risk of mortality [37], however a significant number of COPD patients continue to smoke [38]. Although smoking cessation results in a reduced rate of decline in lung function, disease progression may persist [39].

Influenza vaccination can reduce serious illness and death in patients with COPD [40–43].

Furthermore, a significant reduction in exacerbation rate has also been observed with vaccination versus placebo [40]. Findings from a population-based study suggested that COPD patients, especially the elderly, had a decreased risk of ischemic heart disease when receiving the influenza vaccine over many years [44].

Pulmonary rehabilitation (PR) reduces hyperinflation by promoting lung deflation and better lung ventilation, which is linked to an improved health status and exercise capacity [45]. PR also provides benefits that extend beyond the initial training period, such as: improved survival; improved recovery following hospitalization for exacerbation; reduced perceived intensity of breathlessness; and reduced anxiety and depression [2, 46, 47]. PR programs can significantly reduce the frequency of exacerbations and hospitalization, and the proportion of patients classified as ‘frequent exacerbator’ [48]. Non-pharmacological interventions, such as surgical modes or bronchoscopic modes of lung volume reduction, are also associated with a reduction of exacerbation; however, these interventions are limited to a small number of patients [49].

Encouraging patients to increase their levels of daily activity is also recommended, due to the general beneficial effects of physical exercise [2] and the reported links between lower levels of physical activity in patients with COPD and an increased risk of hospitalization [50, 51] and readmission [52].

### Pharmacological interventions

It must be recognized that many studies presented here were not designed to test for the relative efficacy of treatments in exacerbation prevention, making it difficult to draw conclusions on their effects in at-risk populations. However, the following studies were powered to test for differences in exacerbation rate, and recruited patients at high risk of exacerbation, thus, enriching study populations: POET (1 year) [53]; SPARK (64 weeks) [24]; FLAME (52 weeks) [29]; and INSPIRE (2 years) [54].

### Single bronchodilation versus placebo in the prevention of exacerbations

The two main classes of bronchodilators are β_2_-agonists and muscarinic receptor antagonists. Both classes of bronchodilators improve patients’ ability to breathe by relaxing airway smooth muscle, thereby reducing respiratory muscle activity and dynamic hyperinflation, and improving ventilatory mechanics [55–57]. Exacerbations are mainly triggered by infections associated with small airway inflammation [3], however environmental conditions, such as pollution, may also initiate or amplify these events [2]. The mechanisms by which bronchodilators prevent exacerbations are unclear, yet are thought to include decreased hyperinflation and mechanical stress, decreased mucus production and enhanced mucociliary clearance, the improvement of symptom severity and fluctuation, and potential anti-inflammatory properties [58]. Compared with placebo, the use of tiotropium 18 μg once daily (q.d.) was associated with sustained reductions in lung hyperinflation and inspiratory capacity both at rest and during exercise, contributing to improvements in exertional dyspnea and increased exercise endurance in patients with COPD [57]. Treatment with formoterol 12 μg twice daily (b.i.d.) significantly enhanced mucus clearance compared with tiotropium 18 μg q.d. in patients with mild-to-moderate COPD [59]. AUGMENT demonstrated that treatment with both formoterol 12 μg b.i.d. and aclidinium 400 μg b.i.d. significantly improved dyspnea and health status compared with placebo in patients with moderate-to-severe COPD [60]. Compared with formoterol 12 μg b.i.d., tiotropium 18 μg q.d. demonstrated a superior anti-inflammatory activity profile, significantly reducing the production of superoxide and pro-inflammatory mediators in COPD patients [61].

Traditionally, much of the evidence for the efficacy of LAMAs in exacerbation prevention has come from studies with tiotropium. More recently, there have been similar findings with glycopyrronium, aclidinium and umeclidinium, as well as the LABAs salmeterol and indacaterol. Table 1 summarizes the findings from major trials of these agents with regards to exacerbation prevention, although it should be noted that exacerbations were studied as a secondary endpoint in the majority of cases.

**Table 1:** Overview of key COPD clinical trials comparing single and dual bronchodilator therapies with placebo

Study title	Study design	Duration	Patient population	Treatment arms	*N*	Exacerbation definition	Key exacerbation results (Comparator vs placebo)
*Single BD (LAMA) vs PBO* Tiotropium							
Casaburi et al. (2002) [62]	MC, R, DB, PC	1 year	FEV_1_ ≤ 65% predicted and ≤ 70% FVC	TIO 18 μg q.d. PBO (3:2)	550 371	Complex of respiratory events (cough, wheezing, dyspnea or sputum production) lasting >3 days (generally treated with AB ± oral CS)	• ≥1 exac: 36% vs 42% (14% reduction with TIO; *p* < 0.05) • Increased time to first exac with TIO vs PBO (*p* = 0.011) • Fewer exac events/pt/yr: 0.76 vs 0.95 (20% reduction with TIO; *p* = 0.045) • Fewer hospitalizations for exac: 0.086 vs 0.161 events/pt/yr (47% reduction; *p* = 0.019) • Fewer patients hospitalized for exac: 5.5% vs 9.4% (41% reduction with TIO; *p* < 0.05)
Brusasco et al. (2003) [63] Combined analysis of NCT02172287/NCT02173691	2 x MC, R, DB, DD, PG, PC	6 months	FEV_1_ ≤ 65% predicted and ≤ 70% FVC	TIO 18 μg q.d. PBO SALM 50 μg b.i.d.^a^ (1:1:1)	402 400 405	Complex of respiratory symptoms (new onset or increase in one or more of cough, sputum, dyspnea, wheeze, chest discomfort) lasting at least 3 days and usually associated with therapeutic intervention	• Delayed time to first exac with TIO vs PBO (*p* ≤ 0.01) • Fewer exac/pt/yr: 1.07 vs 1.49 (28% reduction with TIO vs PBO; *p* = 0.025) • Exac days/pt/yr: 17.2 vs 25 (31% reduction with TIO vs PBO; *p* = 0.025) • No significant differences between TIO and PBO in hospital admissions, days in hospital or unscheduled physician visits for exac
Niewoehner et al. (2005) [64] NCT00274547	MC, R, DB, PG, PC	6 months	Moderate-to-severe COPD (FEV_1_ ≤ 60% predicted and ≤ 70% FVC)	TIO 18 μg q.d. PBO (1:1)	914 915	Complex of respiratory symptoms (increase or new-onset) of more than one of cough, sputum, wheezing, dyspnea, or chest tightness with a duration of ≥3 days requiring treatment with AB or systemic CS, hospitalization or both	• ≥1 exac: 27.9% vs 32.3% (OR 0.81; 95% CI 0.66, 0.99; *p* = 0.037) • ≥1 hospitalization for exac: 7.0% vs 9.5% (OR 0.72; 95% CI 0.51, 1.01; *p* = 0.056, NS) • Extended time to first exac (HR 0.83; 95% CI 0.70, 0.98; *p* = 0.028) • Reductions (events/pt/yr) in TIO vs PBO: o Frequency of exac: 0.85 vs 1.05 (*p* = 0.031) o Exac days: 12.6 vs 16.0 (*p* = 0.019) o Unscheduled medical visits: 0.39 vs 0.49 (*p* = 0.019) o Hospitalizations for exac: 0.18 vs 0.25 (*p* = 0.047)
Dusser et al. (2006) [65] MISTRAL	MC, R, DB, PG, PC	1 year	FEV_1_ 30–65% predicted and FEV_1_/FVC ≤ 0.7	TIO 18 μg q.d. PBO (1:1)	500 510	Onset of at least one clinical descriptor (worsening dyspnea, cough or sputum production; appearance of purulent sputum; fever [>38 ºC]; appearance of new chest radiograph abnormality) lasting ≥2 days and requiring dose increase of β_2_-agonists, AB, CS or BD	• ≥1 exac: 49.9% vs 60.3% (17% reduction with TIO; *p* < 0.01) • Fewer exac/pt/yr: 1.57 vs 2.41 (35% reduction with TIO; *p* < 0.001) • TIO reduced exac days by 37% vs PBO (*p* < 0.001) and delayed time to first exac by ~100 days (*p* < 0.001) • ≥1 moderate-to-severe exac: 42.5% vs 53.4% (30% reduction with TIO, *p* < 0.001)
Powrie et al. (2007) [66] NCT00405236	SC, R, DB, PC	1 year	FEV_1_ < 80% predicted; FEV_1_/FVC < 0.7	TIO 18 μg q.d. PBO (1:1)	69 73	Presence for ≥2 consecutive days of increase in any two major symptoms (dyspnea, sputum purulence, sputum volume) or increase in one major and one minor symptom (wheeze, sore throat, cough, symptoms of a common cold)	• ≥1 exac: 43% vs 64% (*p* = 0.01) • Fewer exac/yr: 1.17 vs 2.46 (52% reduction with TIO; *p* = 0.001) • Time to first exac: 236 vs 157 days (*p* = 0.0092) • Fewer exac days: 17.3 vs 34.5 (*p* = 0.002) • No treatment differences for number of treated exac or hospitalizations for exac
Chan et al. (2007) [125] SAFE	MC, R, DB, PG, PC	1 year	Moderate-to- severe COPD (FEV_1_ ≤ 65% predicted and FEV_1_/FVC ≤ 0.7)	TIO 18 μg q.d. PBO (2:1)	608 305	Complex of respiratory symptoms (new-onset or increase in at least one of cough, sputum, sputum purulence, dyspnea, wheeze, chest discomfort) lasting ≥3 days and requiring treatment with AB ± systemic CS	• No statistically significant differences between TIO and PBO: o ≥1 exac: 44.1% vs 41.0% o ≥1 hospitalization for exac: 8.4% vs 8.2% o Exac/pt/yr: 0.88 vs 0.92 o Exac days/pt yr: 16.13 vs 16.19 o Hospitalizations for exac: 0.13 vs 0.15 o Hospitalization days for exac: 1.14 vs 1.16
Tonnel et al. (2008) [67] TIPHON	MC, R, DB, PG, PC	9 months	Mild, moderate or severe COPD (FEV_1_ 20–70% predicted FEV_1_/FVC ≤ 0.7^b^)	TIO 18 μg q.d. PBO (1:1)	266 288	Worsening of COPD (from stable state beyond normal day-to-day variation) that was acute in onset and necessitated a change in regular medication	• ≥1 exac: 38.0% vs 45.1% (*p* = 0.10; NS) • Fewer exac/yr: 1.05 vs 1.83 (43% reduction with TIO; *p* = 0.03) • Fewer exac days/yr: 10.5 vs 20.6 (49% reduction with TIO; *p* = 0.02) • Delayed time to first exac: 201 days vs 181 days (*p* = 0.0081)
Tashkin et al. (2008) [68] NCT00144339 UPLIFT	MC, R, DB, PG, PC	4 years	Moderate-to-very severe COPD (FEV_1_ ≤ 70% predicted and ≤ 70% FVC)	TIO 18 μg q.d. PBO (1:1)	2,987 3,006	Increase in/new onset of more than one respiratory symptom (cough, sputum, sputum purulence, wheeze, or dyspnea) lasting ≥3 days and requiring treatment with AB or systemic CS	• Exac/pt/yr: 0.73 vs 0.85 (RR 0.86; 95% CI 0.81, 0.91; *p* < 0.001) • Exac days/pt/yr: 12.11 vs 13.64 (RR 0.89; 95% CI 0.83, 0.95; *p* = 0.001) • Hospitalizations for exac (no./pt/yr): 0.15 vs 0.16 (RR 0.94; 95% CI 0.82, 1.07; *p* = NS) • Significantly delayed median time to first exac: 16.7 months (95% CI 14.9, 17.9) vs 12.5 months (95% CI 11.5, 13.8) • Significantly delayed time to first hospitalization for exac^c^
Bateman et al. (2010) [70] NCT00387088	MC, R, DB, PG, PC	48 weeks	FEV_1_ ≤ 60% predicted and FEV_1_/FVC ≤ 0.7	TIO 5 μg q.d.^d^ PBO (1:1)	1,989 2,002	Complex of respiratory events/symptoms lasting ≥3 days and requiring treatment with AB and/or systemic CS, or prompting a change in regular medication	• ≥1 exac: 35.3% vs 43.1% (HR 0.693; 95% CI 0.625, 0.769; *p* < 0.0001) • Fewer exac/pt/yr: 0.69 vs 0.87 (RR 0.79; 95% CI 0.72, 0.87; *p* < 0.0001) • Fewer exac requiring hospitalization (pt/yr: 0.12 vs 0.15 (RR 0.81; 95% CI 0.70, 0.93; *p* < 0.005)
Bateman et al. (2010) [69] Combined analysis of NCT00168844/NCT00168831	2 x MC, R, DB, PG, PC	1 year	Moderate-to-severe COPD (FEV_1_ ≤ 60% predicted and ≤ 70% FVC)	TIO 5 μg q.d.^d^ TIO 10 μg q.d.^d^ PBO (1:1)	670 667 653	Respiratory adverse events lasting ≥3 days and requiring treatment with AB ± oral CS ± a significant change in prescribed medication including inhaled BD	• ≥1 exac: 37.2% (TIO 5 μg) and 36.9% (TIO 10 μg) vs 44.1% (PBO) • Exac rate (per pt-yr) o TIO 5 μg vs PBO: OR 0.75 (*p* < 0.01) o TIO 10 μg vs PBO: OR 0.74 (*p* < 0.001) • Time to first exac (days): 160 and 178 vs 86 (both *p* < 0.001) • Hospitalization/pt/yr: 0.12 and 0.16 vs 0.20 (*p* = NS)
Abrahams et al. (2013) [126] NCT00528996	MC, R, DB, PG, PC	24 weeks	FEV_1_ < 80% predicted and FEV_1_/FVC ≤ 0.7	TIO 5 μg q.d.^d^ PBO (1:1) (BEA2810 50, 100 and 200 μg also assessed^a^)	427 429 (Total 2,080)	Complex of respiratory events/symptoms (increased/new onset of ≥2 of: shortness of breath, sputum production, [volume], purulent sputum, cough, wheeze, chest tightness) related to COPD, with a duration of ≥3 days requiring a change in treatment	• No significant difference between TIO and PBO in risk of COPD exac
Glycopyrronium							
D’Urzo et al. (2011) [71] NCT01005901 GLOW1	MC, R, DB, PG, PC	26 weeks	Moderate-to-severe COPD (FEV_1_ ≥ 30% and < 80% predicted; FEV_1_/FVC < 0.7)	GLY 50 μg q.d. PBO (2:1)	552 270	Increase in ≥2 COPD symptoms or worsening of any one major symptom together with a minor symptom over ≥2 consecutive days. AB ± systemic CS (moderate exac), or hospitalization (severe exac)	• Delayed time to first moderate or severe exac by 31% vs PBO (HR, 0.69; 95% CI 0.500, 0.949; *p* = 0.023) • Reduced risk of severe exac leading to hospitalization vs PBO (HR, 0.35; 95% CI 0.141, 0.857; *p* = 0.022) • Reduced the proportion of hospitalizations due to exacs vs PBO: 1.7% vs 4.2% (OR, 0.34; 95% CI 0.129, 0.868; *p* = 0.024)
Kerwin et al. (2012) [72] NCT00929110 GLOW2	MC, R, DB, PG, PC	52 weeks	Moderate-to-severe COPD (FEV_1_ ≥ 30% and < 80% predicted; FEV_1_/FVC < 0.7)	GLY 50 μg q.d. PBO OL TIO 18 μg q.d.^a^ (2:1:1)	529 269 268	Increase in ≥2 COPD symptoms or worsening of any one major symptom together with a minor symptom over ≥2 consecutive days. AB ± systemic CS (moderate exac), or hospitalization (severe exac).	• Risk of time to first moderate-to-severe exac reduced by 34% with GLY vs PBO (HR 0.66; 95% CI 0.520, 0.850; *p* = 0.001) • Rate of moderate-to-severe exac reduced by 34% with GLY vs PBO (RR 0.66; 95% CI 0.496, 0.869; *p* = 0.003) • Exac requiring treatment with systemic CS or AB significantly reduced with GLY vs PBO (OR 0.61 [*p* = 0.006] and OR 0.69 [*p* = 0.026], respectively)
Aclidinium							
Kerwin et al. (2012) [73] NCT00891462 ACCORD COPD I	MC, R, DB, PG, PC	12 weeks	Moderate-to-severe COPD (FEV_1_ ≥ 30% and < 80% predicted; FEV_1_/ FVC < 0.7)	ACL 200 μg b.i.d. ACL 400 μg b.i.d. PBO (1:1:1)	185 190 186	Increase in COPD symptoms over ≥2 consecutive days resulting in medical intervention	• Rate of any exac significantly reduced with ACL 400 μg vs PBO (RR 0.52; *p* = 0.0009) • Trend (not significant) towards reduced rate of moderate-to-severe exac/pt/yr with ACL 200 μg (33%) and ACL 400 μg (34%) vs PBO
Jones et al. (2012) [74] NCT01001494 ATTAIN	MC, R, DB, PG, PC	24 weeks	Moderate-to-severe COPD (FEV_1_ < 80% predicted; FEV_1_/ FVC < 0.7)	ACL 200 μg b.i.d. ACL 400 μg b.i.d. PBO (1:1:1)	280 272 276	Increase in COPD symptoms over ≥2 consecutive days resulting in increased use of short-acting BD ± ICS (mild exac), AB ± systemic CS (moderate exac), or hospitalization (severe exac)	• Rate of any exac significantly reduced with ACL 200 μg and 400 μg vs PBO (RR: 0.72, 95% CI 0.52, 0.99, *p* < 0.05 and RR 0.67, 95% CI 0.48, 0.94, *p* < 0.05, respectively) • Trend (not significant) towards reduced rate of moderate or severe exac with ACL vs PBO (RR 0.74 for ACL 200 μg [*p* = 0.08] and 0.72 for ACL 400 μg [*p* = 0.06])
Umeclidinium							
Donohue et al. (2013) [22] NCT01313650 DB2113373	MC, R, DB, PG, PC	24 weeks	FEV_1_ ≤ 70% predicted and FEV_1_/FVC < 0.7	UMEC/VI 62.5/25 μg q.d.^a^ UMEC 62.5 μg q.d. VI 25 μg q.d.^a^ PBO (3:3:3:2)	413 418 421 280	Acute worsening of symptoms of COPD requiring emergency treatment, hospitalization or use of additional therapy beyond study drug/rescue salbutamol (*e.g.* oral CS and AB)	• Reduced risk of exac with UMEC vs PBO (HR 0.6; 95% CI 0.4, 1.0, *p* < 0.05)
Celli et al. (2014) [75] NCT01313637	MC, R, DB, PG, PC	24 weeks	FEV_1_ ≤ 70% predicted and FEV_1_/FVC < 0.7	UMEC/VI 125/25 μg q.d.^a^ UMEC 125 μg q.d. VI 25 μg q.d.^a^ PBO (3:3:3:2)	403 407 404 275	Acute worsening of symptoms of COPD requiring emergency treatment, hospitalization or use of any therapy beyond study drug/rescue albuterol	• Reduced risk of COPD exac with UMEC vs PBO (HR 0.5; 95% CI 0.3, 0.8, *p* ≤ 0.006)
*Single BD (LABA) vs PBO* Salmeterol							
Stockley et al. (2006) [76]	MC, R, DB, PC	1 year	FEV_1_ < 70% predicted and established history of exacerbations (≥2 in previous year needing treatment with AB and/or oral CS)	SALM 50 μg b.i.d. PBO (1:1)	316 318	Exacerbations were identified using an event-based definition in which a worsening of symptoms required a change in medication. Exacerbations classed as moderate if required treatment with AB +/- oral CS/increase in ICS dose; severe if required hospital admission	• Mean number of moderate/severe exac/year in ITT population was lower with SALM (0.93) vs PBO (1.18); *p* = NS • Mean number of moderate/severe exac/year in PP population was significantly lower with SALM (0.58) vs PBO (0.83); *p* = 0.007
Indacaterol							
Dahl et al. (2010) [77] NCT00393458 INVOLVE	MC, R, DB, DD, PG, PC	1 year	Moderate-to-severe COPD (FEV_1_ < 80% and ≥ 30% predicted and FEV_1_/FVC < 0.7)	IND 300 μg q.d. IND 600 μg q.d. PBO FOR 12 μg b.i.d.^a^ (1:1:1:1)	437 428 432 435	Onset/worsening of more than one respiratory symptom (dyspnea, cough, sputum purulence/volume or wheeze) for >3 consecutive days plus documented proof of intensified treatment (*e.g.* systemic CS, AB or oxygen) ± hospitalization/ER visit	• Exac: 32.8% (IND 300 μg) and 29.3% (IND 600 μg) vs 36.3% (PBO) • Time to first exac improved with IND 300 μg and 600 μg vs PBO: HR 0.77 (95% CI 0.606, 0.975, *p* < 0.05) and 0.69 (95% CI 0.538, 0.882, *p* < 0.05) • RR vs PBO were 0.82 for IND 300 μg (*p* = NS) and 0.74 (0.74, 95% CI 0.56, 0.97, *p* < 0.05) for IND 600 μg
Donohue et al. (2010) [79] NCT00463567 INHANCE	MC, R, DB, PC	26 weeks	Moderate-to-severe COPD	IND 150 μg q.d. IND 300 μg q.d. PBO OL TIO 18 μg q.d.^a^	416 416 418 415	Onset/worsening of one or more respiratory symptoms (dyspnea, cough, sputum purulence/volume, or wheeze) for ≥3 consecutive days, plus intensified treatment (*e.g.*, systemic CS, AB, oxygen) ± hospitalization/ER visit	• Risk of time to first exac reduced vs PBO for IND 150 μg (HR 0.69; 95% CI 0.51, 0.94; *p* = 0.019); numerically reduced risk for IND 300 μg (HR 0.74; 95% CI 0.55, 1.01, *p* = 0.054) • RR of exac vs PBO: 0.67 IND 150 μg (95% CI 0.46, 0.99; *p* = 0.044); for IND 300 μg (0.75, 95% CI 0.51, 1.08, *p* = NS) • Rate of exac/yr: 0.50 and 0.53 vs 0.72 for IND 150 μg, 300 μg vs PBO, respectively
Chapman et al. (2011) [78] NCT00677807 INDORSE	MC, R, DB, PC	26-week extension (52 weeks including core study; *see above*)	Moderate-to-severe COPD (FEV_1_ < 80% and ≥ 30% predicted; and FEV_1_/FVC < 0.7)	IND 150 μg q.d. IND 300 μg q.d. PBO	420 418 425	Onset/worsening of more than one respiratory symptom (dyspnea, cough, sputum purulence/volume, or wheeze) for >3 consecutive days, plus intensified treatment (*e.g.*, systemic CS, AB, oxygen) ± hospitalization/ER visit	• Exac/yr: 0.39 (IND 150 μg; *p* < 0.05) and 0.38 (IND 300 μg; *p* = NS) vs 0.54 (PBO) • RR of exac vs PBO: 0.64 IND 150 μg (95% CI 0.43, 0.96, *p* = 0.029); 0.62 IND 300 μg (95% CI 0.42, 0.92, *p* = 0.018) • Time to first exac: HR (vs PBO): 0.82 (95% CI 0.51, 1.34) IND 150 μg; 0.86 (95% CI 0.53, 1.39) IND 300 μg^e^
*Dual bronchodilation (LAMA/LABA) vs PBO* Aclidinium/formoterol							
Singh et al. (2014) [23] NCT01462942 ACLIFORM-COPD	MC, R, DB, PG, PC/AC	24 weeks	Moderate-to-severe COPD (FEV_1_ < 80% and ≥ 30% predicted and FEV_1_/FVC < 0.7)	A/F 400/12 μg b.i.d. A/F 400/6 μg b.i.d. ACL 400 μg b.i.d.^a^ FOR 12 μg b.i.d.^a^ PBO (2:2:2:2:1)	385 381 385 384 194	HCRU: increase of COPD symptoms during ≥2 consecutive days that require a change in COPD treatment; and EXACT: persistent increase from baseline in total EXACT score of ≥9 points for ≥3 days or ≥12 points for ≥2 days	• HCRU rate: 27% lower with A/F 400/12 μg vs PBO (did not reach significance); RR were 0.73 A/F 400/12 μg and 0.80 A/F 400/6 μg • EXACT rate: significantly lower with A/F 400/12 μg vs PBO (0.71; 95% CI 0.5, 0.9, *p* < 0.05) • No. pts hospitalized for exac was low and similar between treatments
Bateman et al. (2015) [84] Pooled analysis of ACLIFORM-COPD and AUGMENT NCT01462942/NCT01437397	2 x MC, R, DB, PG, PC/AC	24 weeks	Moderate-to-severe COPD (FEV_1_ < 80% and ≥ 30% predicted and FEV_1_/FVC < 0.7)	A/F 400/12 μg b.i.d. A/F 400/6 μg b.i.d.^f^ ACL 400 μg b.i.d.^a^ FOR 12 μg b.i.d.^a^ PBO	723 719 725 723 531	HCRU: increase in COPD symptoms during ≥2 consecutive days that required a change in COPD treatment; and EXACT: persistent increase from baseline in total EXACT score of ≥9 points for ≥3 days or ≥12 points for ≥2 days	• HCRU rate: 24% (RR 0.76; p = NS) and 29% (RR 0.71; p < 0.05) reductions in any and in moderate or severe exac, respectively • Increased time to first exac vs PBO: o Any severity (HR 0.72; 95% CI 0.53, 0.97, *p* < 0.05) o Moderate or severe (HR 0.70; 95% CI 0.51, 0.96, *p* < 0.05) • Results supported by EXACT data: o RR 0.78 (95% CI 0.65, 0.94, *p* < 0.001) o Time to first EXACT exac of any severity (HR 0.79; 95% CI 0.65, 0.95, *p* < 0.05)
Umeclidinium/vilanterol							
Donohue et al. (2013) [22] NCT01313650 DB2113373	MC, R, DB, PG, PC/AC	24 weeks	FEV_1_ ≤ 70% predicted and FEV_1_/FVC < 0.7	UMEC/VI 62.5/25 μg q.d. UMEC 62.5 μg q.d.^a^ VI 25 μg q.d.^a^ PBO (3:3:3:2)	413 418 421 280	Acute worsening of symptoms of COPD requiring emergency treatment, hospitalization or use of additional therapy beyond study drug/rescue salbutamol (*e.g.* oral CS and AB)	• Reduced risk of exac with UMEC/VI vs PBO (HR 0.5; 95% CI 0.3, 0.8, *p* ≤ 0.01)
Celli et al. (2014) [75] NCT01313637	MC, R, DB, PG, PC/AC	24 weeks	FEV_1_ ≤ 70% predicted and FEV_1_/FVC < 0.7	UMEC/VI 125/25 μg q.d. UMEC 125 μg q.d.^a^ VI 25 μg q.d.^a^ PBO (3:3:3:2)	403 407 404 275	Acute worsening of symptoms of COPD requiring emergency treatment, hospitalization or use of any therapy beyond study drug/rescue albuterol	• Reduced risk of COPD exac with UMEC/VI vs PBO (HR 0.4; 95% CI 0.2, 0.6, p ≤ 0.006)

^a^Data not included; ^b^According to 1995 American Thoracic Society criteria; ^c^Hospitalizations occurred in <50% of patients and therefore a median time to first event could not be calculated; ^d^Soft mist formulation delivered via the Respimat^®^ device; ^e^Study was not powered to make comparison; ^f^Data for aclidinium/formoterol 400/6 μg b.i.d. not reported in publication. *AB* antibiotics, *AC* active controlled, *ACCORD COPD I* AClidinium in Chronic Obstructive Respiratory Disease COPD I, *ACL aclidinium; A/F* aclidinium/formoterol, *AUGMENT* Aclidinium/formoterol FUmarate Combination for InvestiGative use in the TreatMENT of Moderate-to-Severe COPD, *ATTAIN* Aclidinium To Treat Airway obstruction In COPD patieNts, *BD* bronchodilators, *b.i.d.* twice daily, *CI* confidence interval, *CS* corticosteroids, *DB* double blind, *DD* double dummy, *ER* emergency room, *exac* exacerbation, *EXACT* EXAcerbations of Chronic pulmonary disease Tool, *FEV*_*1*_ forced expiratory volume in 1 s, *FVC* forced vital capacity, *FOR* formoterol, *GLOW2* GLycopyrronium bromide in COPD airWays clinical Study 2, *GLY* glycopyrronium, *HCRU* Healthcare Resource Utilization, *HR* hazard ratio, *ICS* inhaled corticosteroids, *IND* indacaterol, *INHANCE* INdacaterol [versus tiotropium] to Help Achieve New COPD treatment Excellence, *INVOLVE*: Indacaterol: Value in COPD: Longer Term Validation of Efficacy and Safety, *LABA* long-acting β_2_-agonist, *LAMA* long-acting muscarinic antagonist, *MC* multicenter, *MISTRAL* Mesure de l’Influence de Spiriva^®^ sur les Troubles Respiratoires Aigus à Long terme, *NS* not statistically significant, *OL* open label, *OR* odds ratio, *PBO* placebo, *PC* placebo controlled, *PG* parallel group, *PP* per protocol; *pt* patient, *q.d.* once daily, *R* randomized, *RR* relative risk, *SALM* salmeterol, *SC* single center, *SVC* slow vital capacity, *TIO* tiotropium, *UMEC* umeclidinium, *VI* vilanterol, *UPLIFT*: Understanding Potential Long-Term Impacts on Function with Tiotropium, *yr* year

Most of the 11 studies comparing tiotropium (5 or 10 μg q.d., via the soft-mist inhaler, or 18 μg q.d. via dry-powder inhaler) with placebo reported significant beneficial effects on various exacerbation-related outcomes. In nine studies, the number of exacerbation events per patient per year was significantly lower with tiotropium than placebo [62–70]. Eight studies reported significant delays in the time to first exacerbation with tiotropium versus placebo [62–69], and in six studies the proportion of patients experiencing one or more exacerbations, and the number of exacerbation days per year, were significantly lower with tiotropium than with placebo [62, 64–70]. Only three studies reported significantly lower hospitalizations due to exacerbation (rates, events or proportions of patients) with tiotropium [62, 64, 70]. Glycopyrronium (50 μg q.d.) [71, 72], aclidinium (200 or 400 μg b.i.d. [73, 74], umeclidinium (62.5 μg and 125 μg q.d.) [22, 75], salmeterol (50 μg b.i.d.) [76] and indacaterol (doses ranging from 150–600 μg q.d.) [77–79] have demonstrated similar beneficial effects compared with placebo.

In two pivotal studies, GLOW1 (26 weeks) and GLOW2 (1 year), glycopyrronium (50 μg q.d.) significantly prolonged time to first moderate-to-severe exacerbation versus placebo [71, 72]. In GLOW1, glycopyrronium also significantly reduced the risk of severe COPD exacerbations leading to hospitalization and the proportion of hospitalizations due to COPD exacerbations [71]. In GLOW2, glycopyrronium significantly reduced the rate of moderate-to-severe exacerbations and the number of exacerbations requiring treatment with systemic corticosteroids or antibiotics, versus placebo [72].

In ACCORD (12 weeks) and ATTAIN (24 weeks), aclidinium (200 or 400 μg b.i.d.) significantly reduced the rate of exacerbations of any severity and numerically reduced rates of moderate or severe exacerbations per patient per year compared with placebo [73, 74]. Two 24-week studies examining the efficacy of umeclidinium demonstrated significant reductions in the risk of exacerbations versus placebo [22, 75].

### Comparison of the efficacy of single bronchodilators in the prevention of exacerbations

Only a few head-to-head studies have examined the relative effects of different bronchodilators on exacerbation outcomes (Table 2).

**Table 2:** Overview of key clinical trials comparing single or dual bronchodilator therapies with single bronchodilators

Study title	Study design	Duration	Patient population	Treatment arms	*N*	Exacerbation definition	Key exacerbation results
*Comparison of single BDs* LAMA vs LAMA							
GLOW2 Kerwin et al. (2012) [72] NCT00929110	MC, R, DB, DD, PG, PC, OL	52 weeks	Moderate-to-severe stable COPD (FEV_1_ ≥ 30% and <80% predicted; FEV_1_/ FVC < 0.7)	GLY 50 μg q.d. PBO^a^ OL TIO 18 μg q.d. (2:1:1)	529 269 268	N/A	• Time to first moderate or severe exac: comparable risk reduction for GLY and TIO vs PBO^b^ o 34% risk reduction with GLY vs PBO (HR 0.66; 95% CI 0.520, 0.850, *p* = 0.001) o 39% risk reduction with TIO vs PBO (HR 0.61; 95% CI 0.456, 0.821, *p* = 0.001) • Rate of moderate or severe exac: o 34% with GLY vs PBO (RR 0.66; 95% CI 0.496, 0.869, *p* = 0.003) o *P* = NS for TIO vs PBO (RR 0.80; 95% CI 0.586, 1.105)
LAMA vs LABA or LABA vs LAMA							
Vogelmeier et al. (2011) [53] NCT00563381 POET	MC, R, DB, DD, PG, AC	1 year	Moderate-to-very-severe COPD (FEV_1_ ≤ 70% predicted and FEV_1_/FEV ≤ 0.7) plus a history of exac in the preceding year	TIO 18 μg q.d. SALM 50 μg b.i.d. (1:1)	3,707 3,669	Increase in/onset of more than one symptom of COPD (cough, sputum, dyspnea, wheezing, chest tightness) with at least one lasting ≥3 days and requiring treatment with systemic CS, AB or both (criterion for moderate exac) or hospitalization (criterion for severe exac)	• Time to first exac increased by 42 days with TIO vs SALM (145 days vs 187 days; 17% reduced risk; HR 0.83, 95% CI 0.77, 0.90, *p* < 0.001) • TIO increased time to first severe exacerbation vs SALM (HR 0.72, 95% CI 0.61, 0.85, *p* < 0.001) • TIO significantly reduced risk of moderate and severe exac vs SALM by 14% (HR 0.86, 95% CI 0.79, 0.93, *p* < 0.001) and 28% (HR 0.72, 95% HR, 0.61, 0.85, *p* < 0.001), respectively • TIO vs SALM reduced the annual rate of moderate exac by 7% (0.54 vs 0.59; RR 0.93, 95% CI 0.86, 1.00, *p* < 0.05) and severe exac by 27% (0.09 vs 0.13; RR 0.73, 95% CI 0.66, 0.82, *p* < 0.001) • TIO reduced the risk of exac requiring treatment with CS and or AB (*p* < 0.001)
Decramer et al. (2013) [80] NCT00845728 INVIGORATE	MC, R, blinded, DD, PG, AC	52 weeks	Severe COPD (FEV_1_ 30% and < 50% predicted and FEV_1_/FVC < 0.70 plus and a documented history of ≥ 1 moderate or severe exac in the previous 12 months	IND 150 μg q.d. TIO 18 μg q.d. (1:1)	1,723 1,721	Worsening for ≥2 consecutive days of ≥2 major symptoms (dyspnea, sputum volume or sputum purulence) or worsening of any one major symptom plus one minor symptom (sore throat, colds, fever without other cause, increased cough or increased wheeze)	• Rate of exac^c^: 0.79 with IND and 0.61 with TIO (non-inferiority not met; RR 1.29, *p* = NS) • Annual rate of exac higher with IND vs TIO: 0.90 vs 0.73 (RR 1.24; 95% CI 1.12, 1.37, *p* < 0.0001)^d^ • No treatment difference in rates of exac leading to hospitalization in patients receiving ICS
*Comparison of dual vs single BDs* LAMA/LABA vs LAMA or LABA							
Aaron et al. (2007) [127] ISRCTN29870041	MC, R, DB, PG, PC	52 weeks	Moderate or severe COPD (FEV_1_ < 65% predicted and FEV_1_/FVC < 0.7)	TIO 18 μg q.d. TIO 18 μg q.d. + SALM 50 μg b.i.d. TIO 18 μg q.d. + SFC 50/500 μg b.i.d.^a^	156 148 145	Sustained worsening of patient’s respiratory condition, from stable state and beyond normal day-to-day variations, requiring a change in regular medication^e^	• Pts with ≥1 exac: 64.8% TIO + SALM vs 62.8% with TIO (*p* = NS) • Exac/pt/yr: 1.75 TIO + SALM vs 1.61 TIO (*p* = NS) o IRR vs TIO: 1.09 (95% CI 0.84, 1.40) • Time to first exac: 128 days TIO + SALM vs 130 days TIO (*p* = NS) • No. of hospitalizations for exac: 38 vs 49 (*p* = NS)
Wedzicha et al (2013) [24] NCT01120691 SPARK	MC, R, DB, PG	64 weeks	Severe or very severe COPD (FEV_1_ < 50% predicted and FEV_1_/FVC < 0.7) plus a documented history of ≥ 1 exac in previous 12 months requiring treatment with systemic CS or AB or both	IND/GLY 110/50 μg q.d. GLY 50 μg q.d. OL TIO 18 μg q.d. (1:1:1)	741 741 742	Presence of two major symptoms (dyspnea, sputum volume, sputum purulence) for ≥2 consecutive days or a worsening of one major symptom together with an increase in any one minor symptom (sore throat, cold, fever without other cause, cough, wheeze) for ≥2 consecutive days	• IND/GLY significantly reduced annualized rate of moderate or severe exac by 12% vs GLY (RR 0.88; 95% CI 0.77, 0,99, *p* = 0.038) o 10% reduction vs TIO (RR 0.90; 95% CI 0.79, 1.02, *p* = NS) • Rate of all exac reduced with IND/GLY vs GLY (RR 0.85; 95% CI 0.77, 0.94, *p* = 0.0012) and vs TIO (RR 0.86; 95% CI 0.78, 0.94, *p* = 0.0017)
Maleki-Yazdi et al. (2014) [128] NCT01777334 ZEP117115	MC, R, blinded, DD, PG	24 weeks	Moderate-to-very-severe COPD (FEV_1_ ≤ 70% predicted and FEV_1_/FVC < 0.7) plus mMRC score of ≥ 2	UMEC/VI 62.5/25 μg q.d. TIO 18 μg q.d. (1:1)	454 451	Acute worsening of COPD symptoms requiring use of any treatment beyond study drug or rescue albuterol/salbutamol	• Time to first exac reduced with UMEC/VI vs TIO (HR 0.5; 95% CI 0.3, 1.0, *p* = 0.044)
Decramer et al. (2014) [85] Study 1 (S1) NCT01316900 DB2113360 Study 2 (S2) NCT01316913 DB2113374	2 x MC, R, blinded, DD, PG, AC	24 weeks	Moderate-to-very-severe COPD (FEV_1_ ≤ 70% predicted and FEV_1_/FVC < 0.7) plus mMRC score of ≥ 2	UMEC + VI 125 + 25 μg q.d. UMEC + VI 62.5 + 25 μg q.d. UMEC 125 μg q.d. VI 25 μg q.d. TIO 18 μg q.d.	S1 216 S2 217 S1 212 S2 218 S2 222 S1 209 S1 209 S2 215	Acute worsening of symptoms of COPD requiring the use of any treatment other than study drug or rescue salbutamol	• No significant differences in risk of exac between UMEC + VI vs UMEC, VI or TIO monotherapies. Time to first exac: o S1: UMEC 125 μg + VI 25 μg (HR 1.0 vs TIO; 0.6 vs VI) o S1: UMEC 62.5 μg + VI 25 μg (HR 1.2 vs TIO; 0.7 vs VI) o S2: UMEC 125 μg + VI 25 μg (HR 1.1 vs TIO; 0.6 vs UMEC 125) o S2: UMEC 62.5 μg + VI 25 μg (HR 1.9 vs TIO; 0.1.0 vs UMEC 125)
Buhl et al. (2015) [20] Combined data for NCT01431274 TOnado 1 and NCT01431287 TOnado 2	2 x MC, R, DB, AC, PG	24 weeks	Moderate-to-very-severe COPD (FEV_1_ < 80% predicted and FEV_1_/FVC < 0.7) plus mMRC score of ≥ 2	TIO + OLO 5/5 μg q.d. TIO + OLO 2.5/5 μg q.d. OLO 5 μg q.d. TIO 5 μg q.d. TIO 2.5 μg q.d.	1,029 1,030 1,038 1,033 1,032	N/A	• Trend for improvements in moderate/severe exac with TIO + OLO vs monotherapies^f^ • Risk ratios for o TIO + OLO 5/5 μg vs OLO (0.83; *p* = 0.033); vs TIO 5 μg (0.92; *p* = NS) o TIO + OLO 2.5/5 μg vs OLO (0.69; *p* < 0.0001); vs TIO 2.5 μg and 5 μg (both 0.76; *p* = 0.0021)

^a^Data not included in this table; ^b^A direct comparison between glycopyrronium and tiotropium was not conducted; ^c^Non-inferiority comparison in per protocol population; ^d^Pre-specified superiority comparison in full analysis population; ^e^Defined according to the 2000 Aspen Lung Conference Consensus; ^f^Studies were not designed to assess impact of tiotropium + olodaterol fixed-dose combination on COPD exacerbations. *AB* antibiotics, *AC* active controlled; *BD* bronchodilators, b.i.d., twice daily; *CI* confidence interval, *CS* corticosteroids, *DB* double blind, *DD* double dummy, *exac* exacerbation, *FEV*_*1*_ forced expiratory volume in 1 s, *FVC* forced vital capacity, *GLOW2* GLycopyrronium bromide in COPD airWays clinical Study 2, *GLY* glycopyrronium, *HR* hazard ratio, *ICS* inhaled corticosteroids; *IND* indacaterol, *INVIGORATE* indacaterol: providing opportunity to reengage patients with life, *IRR* incidence rate ratio, *LABA* long-acting β_2_-agonist, *LAMA* long-acting muscarinic antagonist, *MC* multicenter, *mMRC* modified Medical Research Council, *N/A* not available in publication, *NS* not statistically significant, *OL* open label, *OLO* olodaterol, *PBO* placebo, *PC* placebo controlled, *PG* parallel group, *POET-COPD* Prevention of Exacerbations with Tiotropium in COPD, *pt* patient, *q.d.* once daily, *R* randomized, *RR* rate ratio, *SALM* salmeterol, *SFC* salmeterol fluticasone propionate combination, *TIO* tiotropium, *UMEC* umeclidinium, *VI* vilanterol

The first study to specifically test the efficacy of a LABA versus a LAMA in exacerbation prevention was POET, a randomized, double-blind, double-dummy, parallel-group trial in patients with moderate-to-very-severe COPD and a history of exacerbations. Compared with salmeterol (50 μg b.i.d.), tiotropium (18 μg q.d.) delayed the time to first exacerbation and significantly reduced the risk of exacerbation (187 days versus 145, respectively; hazard ratio [HR] 0.83; *p* < 0.001). Tiotropium also significantly prolonged the time to first severe exacerbation (HR 0.72; *p* < 0.001) and reduced the annual rates of severe, and moderate or severe exacerbations versus salmeterol (rate ratios [RR], 0.73 [*p* < 0.001] and 0.89 [*p* = 0.002], respectively) [53]. Similar findings were reported in INVIGORATE, where tiotropium (18 μg q.d.) significantly reduced annualized exacerbation rate versus indacaterol (150 μg q.d.) (0.73 versus 0.90; RR 1.24; *p* < 0.0001) [80].

To date, no direct head-to-head, LAMA versus LAMA studies have been performed. In GLOW2, open-label tiotropium (18 μg q.d.) was included as a reference arm; however, the study was not designed nor powered to test for differences between the two active treatments. Compared with placebo, exacerbation risk was reduced with glycopyrronium (HR 0.66; *p* = 0.001) and with tiotropium (HR 0.61; *p* = 0.001), although no formal comparisons between the two treatments were made [72]. In SPARK, which studied the efficacy of indacaterol/glycopyrronium (IND/GLY 110/50 μg q.d.) compared with glycopyrronium (50 μg q.d.) and tiotropium (18 μg q.d.) in patients with severe-to-very severe COPD and an exacerbation history, the rate of moderate or severe exacerbations was similar between glycopyrronium and tiotropium monotherapies (HR 1.03; *p* = 0.68) [24].

### Dual bronchodilation versus placebo in the prevention of exacerbations

The mechanisms underlying interactions between LABAs and LAMAs have not been fully elucidated. However, β_2_-agonists can amplify muscarinic antagonist-mediated smooth muscle relaxation by modulating cholinergic neurotransmission and decreasing acetylcholine release, and muscarinic antagonists can augment β_2_-agonist-mediated bronchodilation by reducing the bronchoconstrictor effects of acetylcholine [81]. The complimentary mechanisms of action of LABAs and LAMAs elicit additive effects on lung function, and provide a rationale for combining the two agents to optimize bronchodilation. Mechanisms that most likely involve reduced airway resistance, improved inspiratory capacity and reduced hyperinflation may confer benefits in terms of exacerbations [58].

Numerous dual bronchodilators have been developed with this aim in mind. They include once-daily IND/GLY, umeclidinium/vilanterol (UMEC/VI) and tiotropium/olodaterol (TIO/OLO), and twice-daily aclidinium/formoterol (A/F) and glycopyrrolate/formoterol fumarate (GLY/F). In the USA, IND/GLY has been developed for twice-daily use [82]. Table 1 summarizes data from studies comparing the effects of dual bronchodilators on exacerbations with placebo.

Exacerbation risk was significantly reduced relative to placebo in UMEC/VI (62.5/25 and 125/25 μg) studies [22, 75] whereas the effects of A/F were less consistent across different doses (400/12 and 400/6 μg) and exacerbation assessments [23, 83]. In a prespecified analysis of pooled data from ACLIFORM and AUGMENT, the higher dose of A/F significantly reduced the rate of moderate or severe exacerbations compared with placebo, whether defined according to EXAcerbation of Chronic Pulmonary Disease Tool (EXACT) criteria (RR 0.78; *p* < 0.01) [84] or healthcare resource utilization (HRU; RR 0.71; *p* < 0.05) [83]. The higher-dose combination of A/F also prolonged time to first exacerbation of any severity defined according to HRU (HR 0.72; *p* < 0.05) or EXACT (HR 0.79; *p* < 0.05) versus placebo ([83]. There are currently no published data comparing IND/GLY or TIO/OLO with placebo [23, 83].

### Dual versus single bronchodilation in the prevention of exacerbations

As shown in Table 2, LABA/LAMAs can improve exacerbation outcomes compared with monotherapy, although not all studies were designed for this objective, and the results are variable. In SPARK, an exacerbation study, the annualized rate of moderate or severe exacerbations was significantly lower with IND/GLY versus glycopyrronium (primary endpoint, RR 0.88; *p* = 0.038), and rates of all exacerbations (mild, moderate or severe) were significantly lower with IND/GLY versus either glycopyrronium (RR 0.85; *p* = 0.0012) or tiotropium (RR 0.86; *p* = 0.0017) [24].

By contrast, in a 24-week study not designed for studying exacerbation prevention, UMEC/VI (125/25 μg q.d.) conferred no significant benefit for exacerbation risk compared with individual monotherapies or with tiotropium (18 μg q.d.) in patients with moderate-to-very severe COPD [85]. The results of the ongoing 52-week DYNAGITO trial (NCT02296138), comparing the annualized rate of moderate-to-severe COPD exacerbations (primary endpoint) with TIO/OLO (5/5 μg q.d.) versus tiotropium (5 μg q.d.) in patients with severe-to-very severe COPD, will therefore be of interest [86].

### Single bronchodilation versus ICS/LABA combinations in the prevention of exacerbations

Of the six ICS/LABA versus bronchodilator monotherapy studies, only two demonstrated significant benefits on exacerbations (Table 3) [87, 88]. It should be noted that these studies were not all designed to compare the effects of an ICS/LABA with single bronchodilation (but with placebo), and exacerbation endpoints were often secondary or exploratory.

**Table 3:** Overview of key clinical trials comparing single or dual bronchodilator therapies with ICS/LABA combination therapy

Study title	Study design	Duration	Patient population	Treatment arms	*N*	Exacerbation definition	Key exacerbation results
*Single BD vs ICS/LABA* LABA: salmeterol							
Calverley et al. (2007) [87] NCT00268216 TORCH	MC, R, DB, PG, PC, AC	3 years	FEV_1_ < 60% predicted and FEV_1_/ FVC ≤ 0.7	SALM 50 μg b.i.d. FP 500 μg b.i.d. SFC 500/50 μg b.i.d. PBO^a^	1,542 1,551 1,546 1,545	Symptomatic deterioration requiring treatment with AB agents, systemic CS, hospitalization or a combination of these	• Annual rate of moderate or severe exac: 0.97 (SALM), 0.93 (FP), 0.85 (FP/SALM), 1.13 (PBO) • Combination therapy reduced the rate of moderate or severe exac; RR: o FP/SALM vs SALM: 0.88 (95% CI 0.81, 0.95, *p* = 0.002) o FP/SALM vs FP: 0.91 (95% CI 0.84, 0.99, *p* = 0.02) • Hospitalization for exac did not differ significantly between FP/SALM and monotherapies
Ohar et al. (2014) [129] NCT01110200 ADC113874	MC, R, DB, PG, AC	26 weeks	FEV_1_ < 70% predicted and FEV_1_/ FVC < 0.7 plus recent (≤ 14 days) history of exac requiring hospitalization for ≤ 10 days; ER observation for ≥ 24 h during which OCS/ OCS + AB administered; or physician’s office/ER visit of < 24 h with OCS/OCS + AB and 6-month history of exac-related hospitalization	SALM 50 μg b.i.d. SFC 250/50 μg b.i.d. (1:1)	325 314	Worsening for ≥2 documented consecutive days of at least two of: dyspnea, sputum volume, sputum purulence, or at least one of these combined with sore throat, cold symptoms, fever or increased cough or wheeze	• No significant difference between FP/SALM vs SALM in rates of recurrent severe (ratio 0.92; 95% CI 0.58, 1.45) or moderate/severe (ratio 0.82; 95% CI 0.64, 1.06) exac • No difference between FP/SALM vs SALM in time to first moderate/severe exac (HR 0.83; 95% CI 0.63, 1.09) • Annualized exac rates in patient subgroup^b^ lower with FP/SALM (1.54) vs SALM (2.28); ratio 0.68 (95% CI 0.47, 0.97)
LABA: formoterol							
Calverley et al. (2010) [130] NCT476099	MC, R, DB, DD, PG, AC	48 weeks	Severe stable COPD (FEV_1_ 30–50% predicted and FEV_1_/FVC ≤ 0.7) plus ≥ 1 exac requiring medical intervention (OCS and/or AB and/or ER visit and/or hospitalization) within 2–12 months before screening and to be clinically stable for 2 months before study entry	FOR 12 μg b.i.d. BDP/FOR 200/12 μg b.i.d. BUD/FOR 400/12 μg b.i.d. (1:1:1)	239 237 242	Need for treatment with OCS and/or AB and/or visit/admission to hospital.	• ≥1 exac and mean rate/pt/yr similar between groups; corresponding data were o BDP/FOR: 27.6% and 0.414 o BUD/FOR: 26.9% and 0.423 o FOR: 28.3% and 0.431 • Hospitalizations for exac: 5.6% for BDP/FOR, 2.9% for BUD/FOR and 3.4% for FOR (*p* < 0.001 and *p* = 0.008 vs BDP/FOR, respectively)
LABA: vilanterol							
Dransfield et al. (2013) [88] Pooled analysis Study 1 NCT01009463 HZC102871 Study 2 NCT01017952 HZC102970	2 x MC, R, DB, PG, AC	1 year	FEV_1_ ≤ 70% predicted and FEV_1_/FEV ≤ 0.7 plus a documented history of ≥ 1 exac requiring treatment (systemic/OCS/AB/hospitalization) in the preceding year	VI 25 μg q.d. FF/VI 50/25 μg q.d. FF/VI 100/25 μg q.d. FF/VI 200/25 μg q.d. (1:1:1:1)	818 820 806 811	Worsening symptoms of COPD (≥2 consecutive days) necessitating treatment with OCS or AB or both; severe exac were similar events that necessitated hospital admission	• Mean annual rate of moderate and severe exac was significantly lower with FF/VI vs VI alone; yearly ratios vs VI were o 0.8 (*p* = 0.0398) FF/VI 50/25 μg o 0.8 (*p* = 0.0244) FF/VI 100/25 μg o 0.7 (*p* = 0.0004) FF/VI 200/25 μg • Time to first moderate or severe exac longer with FF/VI 100/25 and 200/25 μg vs VI o HR 0.8 (95% CI, 0.7, 1.0, *p* = 0.0365) and 0.7 (95% CI, 0.5, 0.8, *p* = 0.0001), respectively • Exac necessitating treatment with CS significantly lower with FF/VI vs VI alone (*p* < 0.05 for 100/25 μg and *p* = 0.0009 for 200/25 μg)
Martinez et al. 2016 [89] NCT01313676 SUMMIT (post hoc analysis)	MC, R, DB, PG, PC	Event-driven, mortality^c^	Moderate COPD (FEV_1_ ≥ 50– ≤ 70% predicted; FEV_1_/FEV ≤ 0.7) and a history of/multiple risk factors for CV disease^d^	FF/VI 100/25 μg q.d. FF 100 μg q.d. VI 25 μg q.d. PBO^a^	4121 4135 4118 4111	Moderate exac: treated with AB and/or systemic CS; severe exac: required hospitalization	• % reduction in moderate/severe exacerbations compared with PBO: 12% (95% CI 4, 19) for FF; 10% (95% CI 2, 18) for VI; and 29% (95% CI 22, 35) for FF/VI • % reduction in exacerbations requiring hospital admissions compared with PBO: 18% (95% CI 3, 31) for FF; 20% (95% CI 5, 32) for VI; and 27% (95% CI 13, 39) for FF/VI • FF/VI reduced the rate of moderate/severe exacerbations by 19% vs FF (95% CI 12, 26, *p* < 0.001) and by 21% vs VI (95% CI 14, 28, *p* < 0.001) • FF/VI reduced the % of exacerbations requiring hospital admissions by 11% vs FF (95% CI –6, 25, *p* = 0.204) and by 9% vs VI (95% CI –8, 25, *p* = 0.282)
LAMA: tiotropium							
Wedzicha et al. (2008) [54] NCT00361959 INSPIRE	MC, R, DB, DD, PG	2 years	Severe and very severe COPD (FEV_1_ < 50% predicted) and mMRC score ≥ 2	TIO 18 μg q.d. SFC 500/50 μg b.i.d.	665 658	Defined by HCRU: episodes that required treatment with OCS and/or AB or hospitalization	• No difference in overall rate between FP/SALM (1.28/yr) and TIO (1.32/yr) • Exac requiring AB with FP/SALM vs TIO: 0.97 vs 0.82/yr (*p* = 0.028) • Exac requiring systemic CS with FP/SALM vs TIO: 0.69 vs 0.85/yr (*p* = 0.039) • Hospitalizations: 16% with FP/SALM vs 13% with TIO (*p* = NS)
Study title	Study design	Duration	Patient population	Treatment arms	*N*	Exacerbation definition	Key exacerbation results
*Dual BD vs ICS/LABA* Indacaterol/glycopyrronium							
Bateman et al. and Banerji et al. (2014) [90, 131] NCT01315249 ILLUMINATE (post hoc analysis)	MC, R, DB, DD, PG	26 weeks	Moderate-to-severe COPD (FEV_1_ ≥ 40%– < 80% predicted and FEV_1_/FEV < 0.7)	IND/GLY 110/50 μg q.d. SFC 500/50 μg b.i.d.	258 264	Defined by modified Anthonisen criteria^e^ (increased dyspnea, sputum production and sputum purulence)	• No significant difference between treatments; RR (IND/GLY vs FP/SALM) of moderate/severe exac: 0.80 (95% CI 0.41, 1.56) and all exac: 0.69 (95% CI 0.44, 1.07) • IND/GLY reduced risk of time to first exac by 35% vs FP/SALM (HR 0.65; 95% CI 0.44, 0.96, *p* = 0.03)
Zhong et al. (2015) [28] NCT01709903 LANTERN	MC, R, DB, DD, PG	26 weeks	Moderate-to-severe COPD (FEV_1_ ≥ 30– < 80% predicted and FEV_1_/FEV < 0.7), mMRC score ≥ 2 and history of ≤ 1 exac in the previous year	IND/GLY 110/50 μg q.d. SFC 500/50 μg b.i.d.	372 372	Worsening of symptoms captured via eDiary; defined by Anthonisen criteria^e^. Moderate exac: requiring treatment with systemic CS and/or AB; severe exac: requiring hospitalization/ER visit > 24 hours	• Annualized rate of moderate or severe exac significantly lower with IND/GLY vs FP/SALM (31% reduction; *p* = 0.048) • IND/GLY prolonged time to first moderate or severe exac by 35% (*p* = 0.028) • In patients with a history of moderate or severe exac, annualized rate
Wedzicha et al. (2016) [29] NCT01782326 FLAME	MC, R, DB, DD, PG, NI	52 weeks	Moderate-to-very severe COPD (FEV_1_ ≥ 25– < 60% predicted and FEV_1_/FEV < 0.7), mMRC score ≥ 2 and documented history of ≥ 1 exac treated with systemic CS and/or AB in previous year	IND/GLY 110/50 μg q.d. SFC 500/50 μg b.i.d.	1,680 1,682	Defined according to Anthonisen criteria^e^. Categorized as mild (worsening of symptoms for >2 consecutive days but not requiring treatment), moderate (treated with systemic CS and/or AB) or severe (requiring hospital \admission/ER visit of >24 h plus systemic CS and/or AB)	• Annual rate of all exac: IND/GLY (3.59) was non-inferior to FP/SALM (4.03): representing an 11% lower rate (RR, 0.89, 95% CI 0.83, 0.96, *p* = 0.003) • IND/GLY showed superiority to FP/SALM as the upper limits of the 95% CIs for the primary endpoint RRs were less than 1 • IND/GLY had a longer time to first exac than the FP/SALM group (median, 71 days [95% CI 60, 82] vs. 51 days [95% CI 46, 57]: HR 0.84 (95% CI 0.78, 0.91, representing a 16% lower risk; p < 0.001)
Aclidinium/formoterol							
Vogelmeier et al. (2015) [132] NCT01908140 AFFIRM	MC, R, DB, DD, AC	24 weeks	Symptomatic pts with FEV_1_ < 80%, FEV_1_/FVC < 0.7 and CAT ≥ 10	A/F 400/12 μg b.i.d. SFC 500/50 μg b.i.d.	468 463	Defined by HCRU or identified using EXACT	• ≥1 exac: comparable between treatment groups: o HCRU: 15.8% (A/F) vs 16.6% (FP/SALM); OR 0.95 o EXACT: 37.8% (A/F) vs 39.5% (FP/SALM); OR 0.94
Umeclidinium/vilanterol							
Donohue et al. (2015) [26] Study 1 NCT01817764 DB2114930 Study 2 NCT01879410 DB2114951	MC, R, DB, DD, PG	12 weeks	Moderate-to-severe COPD (FEV_1_ ≥ 30– ≤ 70% predicted), mMRC score ≥ 2, no exacerbations in the previous year	UMEC/VI 62.5/25 μg q.d. SFC 250/50 μg b.i.d.	353 and 349 353 and 348	Captured only as a safety event. Defined as an acute worsening of COPD symptoms requiring use of AB, systemic CS, and/or emergency treatment or hospitalization	NCT01817764 • Exac rate was the same in each treatment group: o 3% (UMEC/VI) vs 3% (FP/SALM) NCT01879410 • Exac rate was the same in each treatment group: o 3% (UMEC/VI) vs 3% (FP/SALM)
Singh et al. (2015) [92] NCT01822899	MC, R, DB, DD, PG	12 weeks	Moderate-to-severe COPD (FEV_1_ ≥ 30– ≤ 70% predicted and FEV_1_/FVC < 0.7), mMRC score ≥ 2, no exacerbations in the previous year	UMEC/VI 62.5/25 μg q.d. SFC 500/50 μg b.i.d.	358 358	Captured only as a safety event. Not defined	• Exac rate was similar between treatment groups: o 2% (UMEC/VI) vs <1% (FP/SALM)
Tiotropium/olodaterol							
Beeh et al. (2016) [133] NCT01969721 ENERGITO	MC, R, DB, DD, PG	12 weeks	Moderate-to-severe COPD (FEV_1_ ≥ 30– < 80% predicted and FEV_1_/FEV < 0.7), no exacerbations in the previous 3 months	TIO/OLO 5/5 μg q.d. TIO/OLO 5/2.5 μg q.d. SFC 500/50 μg b.i.d. SFC 250/50 μg b.i.d.	221 215 219 212	Captured only as a safety event as ‘COPD worsening’	• Exac rate was similar among each of the high- and low-dose groups: o 9.0% (TIO/OLO 5/5 μg) and 8.7% (FP/SALM 500/50 μg) o 5.6% (TIO/OLO 5/2.5 μg) and 4.2% (FP/SALM 250/50 μg)

^a^Data not included in table; ^b^Subgroup of 373 patients with baseline post-bronchodilator % predicted FEV_1_ ≥ 30% and history of prior ICS; ^c^Patients expected to contribute 15–44 months of study time; ^d^For patients aged ≥40 years: any one of established coronary artery disease, established peripheral vascular disease, previous stroke, previous myocardial infarction or diabetes mellitus with target organ disease and for patients aged ≥60 years, any one of those for ≥40 years of age or two of the following: treatment for hypercholesterolemia, hypertension, diabetes mellitus or peripheral vascular disease; ^e^Anthonisen NR et al. Ann Intern Med 1987;106:196–204. *AB* antibiotics, *AC* active controlled, *A/F* aclidinium/formoterol, *BD* bronchodilator, *BDP* beclomethasone/formoterol; b.i.d. twice daily, *BUD/FF* budesonide/formoterol, *CAT* COPD Assessment Test, *CI* confidence interval, *CS* corticosteroids, *CV* cardiovascular, *DB* double blind, *DD* double dummy, *ER* emergency room, *exac* exacerbation(s), *FEV*_*1*_ forced expiratory volume in 1 s, *FVC* forced vital capacity, *EXACT* EXAcerbations of Chronic pulmonary disease Tool, *FF* fluticasone furoate, *FOR* formoterol, *FP* fluticasone propionate, *GLY* glycopyrronium, *HCRU* healthcare resource utilization, *HR* hazard ratio, *ICS* inhaled corticosteroids, *IND* indacaterol, *INSPIRE* Investigating New Standards for Prophylaxis in Reducing Exacerbations, *LABA* long-acting β_2_-agonist, *LAMA* long-acting muscarinic antagonist, *MC* multicenter, *mMRC* modified Medical Research Council, *NI* non-inferiority, *NS* not statistically significant, *OCS* oral corticosteroids, *OLO* olodaterol, *OR* odds ratio, *PBO* placebo, *PC* placebo controlled, *PG* parallel group, *q.d.* once daily, *R* randomized, *RR* rate ratio, *SALM* salmeterol; SFC. Salmeterol/fluticasone propionate combination, *SUMMIT* Study to Understand Mortality and Morbidity in COPD, *TIO* tiotropium, *TORCH* Towards a Revolution in COPD Health, *UMEC* umeclidinium, *VI* vilanterol, *yr* year

One of the most robust studies was TORCH (3 years), which studied deaths (any cause) as the primary outcome, as well as exacerbation frequency between treatments. In TORCH, salmeterol/fluticasone propionate combination (SFC 50/500 μg b.i.d.) significantly reduced the annual rate of moderate or severe exacerbations compared with salmeterol (50 μg b.i.d.; RR 0.88; *p* = 0.002) [87].

In a pooled analysis of two 1-year trials, in which the primary endpoint was the yearly rate of moderate and severe exacerbations, fluticasone furoate/vilanterol (FF/VI; 50/25 μg, 100/25 μg and 200/25 μg q.d.) significantly reduced the rate of moderate or severe exacerbations compared with vilanterol (25 μg q.d.; *p* < 0.05 for all three doses) [88]. FF/VI (100/25 μg and 200/25 μg q.d.) also significantly prolonged time to first moderate or severe exacerbation versus vilanterol monotherapy (HR 0.8; *p* = 0.0365 and HR 0.7; *p* = 0.0001, respectively), and significantly reduced the number of exacerbations requiring systemic corticosteroids (*p* < 0.05 and *p* = 0.0009, respectively) [88].

In a post-factorial analysis of SUMMIT, FF/VI (100/25 μg q.d.) significantly reduced the rate of moderate or severe exacerbations versus fluticasone (100 μg q.d.; *p* < 0.001) and versus vilanterol (25 μg q.d.; *p* < 0.001) [89].

Only INSPIRE has compared an ICS/LABA with LAMA monotherapy in exacerbation prevention [54]. No significant difference was observed between SFC (50/500 μg b.i.d.) and tiotropium (18 μg q.d.) in HRU exacerbation rate (1.28 and 1.32, respectively) in this study [54].

### Dual bronchodilation versus ICS/LABA combinations in the prevention of exacerbations

Exacerbation data are available from a number of studies comparing a LABA/LAMA with an ICS/LABA (Table 3). In a post-hoc analysis of ILLUMINATE (26 weeks), IND/GLY (110/50 μg q.d.) significantly reduced the time to first exacerbation (HR 0.65; *p* = 0.03) versus SFC (50/500 μg b.i.d.), in patients with moderate–to-severe COPD and no exacerbations in the previous year [90]. Likewise, in LANTERN (26 weeks), IND/GLY (110/50 μg q.d.) significantly reduced the rate of moderate or severe exacerbations (RR 0.69; *p* = 0.048) compared with SFC (50/500 μg b.i.d.), in patients with moderate-to-severe COPD and ≤1 exacerbations in the previous year [28]. In a post-hoc analysis of pooled data from LANTERN and ILLUMINATE, the annualized rate of moderate or severe exacerbations was significantly lower with IND/GLY versus SFC, in both the whole population (*p* = 0.02) and in subgroups of patients classified as either GOLD Group B (*p* = 0.16) or GOLD Group D (*p* = 0.05; patients were classified according to GOLD 2009 and 2010 for ILLUMINATE and LANTERN, respectively). Furthermore, IND/GLY delayed the time to first moderate or severe exacerbation compared with SFC in the overall population and in GOLD Group B and GOLD Group D subgroups [91].

The most recent study comparing a LABA/LAMA with an ICS/LABA was FLAME, which specifically studied the differences in exacerbations between IND/GLY (110/50 μg q.d) and SFC (50/500 μg b.i.d.) as the primary outcome, and included an enriched patient population at high risk of exacerbation (≥1 exacerbation in the previous year) [29]. Compared with SFC, IND/GLY significantly reduced the rate of all COPD exacerbations (RR 0.89; *p* = 0.003), and the rate of moderate or severe exacerbations (RR 0.83; *p* < 0.001). Additionally, IND/GLY significantly prolonged the time to first exacerbation (HR 0.84; *p* < 0.001). The time to first moderate or severe exacerbation (HR 0.78; *p* < 0.001), and time to the first severe exacerbation (HR 0.81; *p* = 0.046) were also significantly prolonged with IND/GLY versus SFC. Treatment benefit or time to first exacerbation was detected as early as 4 weeks. Compared with SFC, IND/GLY numerically, but not significantly, reduced the rate of exacerbations in patients with a history of ≥2 exacerbations in the previous year, (19% of the patient population). However, it is worth noting that this was a subgroup analysis and FLAME was not powered to detect treatment differences in subgroups [29].

In a 24-week trial comparing A/F (400/12 μg b.i.d.) with SFC (50/500 μg b.i.d), a similar proportion of patients experienced at least one exacerbation in the A/F and SFC groups, regardless of exacerbation definition (HRU or EXACT criteria) [27]. *However, the study was not designed to test* exacerbations.

To date, there have been no long-term studies examining the effects of UMEC/VI or TIO/OLO on exacerbations in at-risk patients. Two 12-week studies comparing the efficacy of UMEC/VI (62/25 μg q.d.) and SFC (50/250 μg b.i.d.) captured exacerbations as safety data and did not perform statistical testing [26, 88]. Likewise, in ENERGITO (a 12-week, randomized, double-blind, four-treatment, crossover study comparing the efficacy of TIO/OLO [5/5 μg and 5/2.5 μg q.d.] with SFC [50/500 μg and 50/250 μg b.i.d.]), exacerbations were captured as safety data (reported as an adverse event of ‘COPD worsening’) [92]. Further long-term studies comparing UMEC/VI and TIO/OLO are required.

### Triple therapy in the prevention of exacerbations

Evidence for the efficacy of triple therapy (ICS/LABA/LAMA) in exacerbation prevention is currently limited. Nevertheless, in TRILOGY (1 year), the adjusted annual rate of moderate-to-severe exacerbations was significantly reduced following step-up to triple therapy (beclomethasone dipropionate/formoterol fumarate/glycopyrronium bromide [BDP/FF/GB] 100/6/12.5 μg two actuations b.i.d.) compared with continuing on BDP/FF 100/6 μg two actuations b.i.d. (0.41 versus 0.53, respectively; RR 0.77; *p* = 0.005) [93].

Similarly, TRINITY (1 year) compared the fixed-dose combination [FDC] of BDP/FF/GB (100/6/12.5 μg b.i.d.) with a free combination of the same agents and LAMA monotherapy (tiotropium 18 μg q.d.) in patients with severe–to-very severe COPD. Compared with TIO, BDP/FF/GB FDC significantly reduced the rate of moderate/severe exacerbations (0.46 versus 0.57, respectively; RR 0.80; *p* = 0.0025). BDP/FF/GB and BDP/FF + TIO showed a similar effect on moderate/severe exacerbations (RR 1.01) [94]. Findings are eagerly anticipated from the ongoing InforMing the PAthway of COPD Treatment (IMPACT) study, comparing the rate of moderate and severe exacerbations between FF/UMEC/VI and FF/VI or UMEC/VI over 52-weeks in symptomatic COPD patients with an exacerbation in the previous 12 months [95].

## Other Pharmacological Treatments In The Prevention Of Exacerbations

### Mucolytics in the prevention of exacerbations

Mucolytics are oral medicines designed to reduce mucus and sputum viscosity, thereby making it easier for patients to cough up mucus and clear it from the airways [96]. The current GOLD recommendations recognize that regular use of mucolytics may be beneficial in patients not receiving ICS, to reduce exacerbations and improve health status [2].

The antioxidant N-acetylcysteine (NAC; 600 mg/day), is recommended by the American College of Chest Physicians and Canadian Thoracic Society for patients with moderate-to-severe COPD and a history of two or more exacerbations in the previous 2 years [97]. However, NAC 600 mg/day is currently not approved for use in the USA. In a 6-month study, the number of exacerbations was reduced by 41% with standard therapy plus NAC versus standard therapy alone, and fewer patients experienced at least one exacerbation (46 versus 63 patients, respectively) [98]. In PANTHEON (1 year), NAC (600 mg/b.i.d.) significantly reduced the annual rate of exacerbations versus placebo (1.16 versus 1.49 exacerbations per patient-year; *p* = 0.0011) [99]. By contrast, in BRONCUS (3 year), a randomized, placebo-controlled study, there was no difference between NAC (600 mg/day) and placebo in the number of exacerbations per year (a primary outcome, [1.25 versus 1.29; *p* = 0.85]), although sub-group analyses suggested that NAC might have reduced exacerbation rate in patients not receiving ICS treatment [100]. In a 2015 meta-analysis of 13 studies (N = 4155), NAC significantly reduced the relative risk of exacerbations in patients with COPD and/or chronic bronchitis. The authors concluded that a dose of 600 mg b.i.d. should be used in patients who have both chronic bronchitis and COPD, whereas the standard 600 mg/day dose should suffice for patients who have chronic bronchitis alone [101]. Accordingly, one study showed that NAC (600 mg b.i.d.) was more effective than placebo in reducing exacerbation risk and prolonging time to first exacerbation in high-risk patients (GOLD Groups C and D, according to the 2011 GOLD recommendations), but not low-risk patients [102].

Two studies have examined the effects of erdosteine on exacerbations. EQUALIFE, an 8-month, randomized, double-blind trial, demonstrated that patients taking erdosteine (300 mg b.i.d.) had significantly fewer exacerbations and spent fewer days in hospital than those on placebo [103]. More recently, Moretti et al demonstrated that 10-day treatment with erdosteine (900 mg/day) was associated with a 39% lower risk of exacerbations in the 2 months post-discharge and a significant delay in time to first exacerbation at post-discharge days 30 (*p* = 0.009) and 60 (*p* = 0.075) compared with placebo, in patients hospitalized following acute exacerbation [104]. In a 6-month, randomized trial, a significantly higher proportion of patients experienced no exacerbations when treated with continuous carbocysteine lysine salt monohydrate (SCMC-Lys; 2.7 g q.d.) compared with placebo (*p* < 0.001) [105]. Similarly, in PEACE (1 year), the annualized rate of exacerbations was significantly lower with carbocysteine (1500 mg/day) than with placebo, representing a 25% reduction in risk (*p* = 0.004) [106].

### Phosphodiesterase-4 inhibitors in the prevention of exacerbations

Phosphodiesterase-4 (PDE-4) inhibitors can inactivate immune and inflammatory cells by blocking the metabolism of cyclic adenosine monophosphate (cAMP) [107]. GOLD recommends addition of the selective, long-acting PDE-4 inhibitor roflumilast to a ICS/LABA/LAMA in GOLD Group D patients who continue to experience exacerbations despite triple therapy, particularly patients with a forced expiratory volume in one second (FEV_1_) <50% predicted, chronic bronchitis, and ≥1 hospitalization for an exacerbation in the previous year [2].

A meta-analysis of 13 studies suggested that roflumilast (500 μg q.d.) was more effective than placebo in reducing the rate of acute exacerbations (*p* < 0.001) [107]. In REACT, roflumilast (500 μg q.d.) reduced the rate of moderate-to-severe exacerbations by 13.2% versus placebo in patients with severe COPD, chronic bronchitis and at risk of frequent and severe exacerbations, and receiving ICS/LABA treatment with or without tiotropium (Poisson regression analysis, *p* = 0.0529) [108]. In a post-hoc analysis of RE(2)SPOND (52 weeks), roflumilast (500 μg q.d.) significantly reduced the rate of moderate or severe exacerbations versus placebo in patients with severe-to-very severe COPD and chronic bronchitis, a history of >3 exacerbations and/or ≥1 hospitalizations in the prior year [109]. Roflumilast is indicated as a maintenance treatment (added on to bronchodilator therapy) in adults with severe COPD associated with chronic bronchitis, and a history of frequent exacerbations [110].

### *Macrolides* in the prevention of exacerbations

Macrolides are antibiotics with antimicrobial, anti-inflammatory, and immunomodulating effects. GOLD 2017 recommends the addition of a macrolide to an ICS/LABA/LAMA regimen in GOLD Group D patients who are former smokers and continue to suffer exacerbations despite triple therapy [2]. In COPD, the best studied macrolide is azithromycin. COLUMBUS (1 year) demonstrated that exacerbation rate was significantly reduced with azithromycin (500 mg three times/week), compared with placebo (odds ratio = 0.58, *p* = 0.001), in patients with COPD who had received treatment for ≥3 exacerbations in the previous year despite optimal inhalation therapy [111]. Similar findings were reported in a 1-year, randomized, controlled trial in patients with COPD at risk for exacerbations, where azithromycin (250 mg q.d.) significantly delayed median time to first exacerbation (266 versus 174 days; *p* < 0.001), and significantly reduced the frequency of exacerbations (1.48 versus 1.83; *p* = 0.01) versus placebo [112]. Similar results have been reported in other, smaller studies comparing erythromycin and placebo [113, 114].

#### Appropriate use of ICS

Much evidence supports the use of ICS in patients with persistent asthma, yet the role of ICS in preventing exacerbations of COPD is less clear [19, 115]. Various methodological issues in trial design and/or statistical analysis affect results and make subsequent study interpretation difficult [116].

However, data are emerging to suggest that raised blood or sputum eosinophil levels could predict a positive response (i.e. a reduction in exacerbations) to ICS/LABA versus LABA monotherapy, or predict any deleterious effects ICS withdrawal may have [117–121].

The potential association between this ‘eosinophilic phenotype’ and a positive response to ICS requires further investigation, as the effect has not been consistent across studies within the same analysis [118], and may only be present in patients with a history of ≥2 exacerbations in addition to raised eosinophil levels [121]. Prospective analysis of FLAME (which excluded patients with a previous diagnosis of asthma and/or a blood eosinophil count >600/mm^3^), demonstrated that IND/GLY was superior to SFC in reducing the rate of moderate-to-severe exacerbations, regardless of baseline eosinophil levels [29]. Thus, further studies are needed before any recommendations can be made regarding the potential use of ICS in specific sub-populations.

## Proposed Treatment Paradigm

**Fig. 1:**
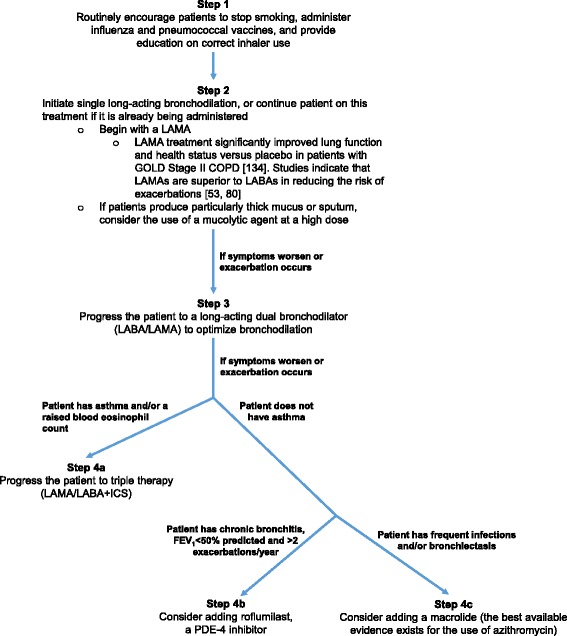
A proposed treatment algorithm for the treatment of chronic obstructive pulmonary disease (COPD). FEV_1_, forced expiratory volume in 1 s; GOLD, Global Initiative for Chronic Obstructive Lung Disease; ICS, inhaled corticosteroids; LABA, long-acting β_2_-agonist; LAMA, long-acting muscarinic antagonist; PDE-4, phosphodiesterase-4 [134]

Figure 1 shows a proposed treatment paradigm for exacerbation prevention based on the evidence presented and centered on optimal use of bronchodilation, which has previously been published [122–124].

## Conclusions

Not only do COPD exacerbations negatively impact the underlying disease course, but they also have a detrimental effect on patients’ lives, resulting in lung function decline, increased risk of mortality and poor health status. While there has been a tendency to recommend the use of ICS for patients at high risk of exacerbations, ICS are associated with a myriad of side effects such as pneumonia. However, (and as recognized in the updated GOLD strategy document) evidence is emerging that suggests there may be more appropriate treatment strategies for many at risk patients, including a LABA/LAMA combination.

The proposed treatment paradigm for exacerbation prevention is centered on optimizing bronchodilation as an initial pharmacological step, first with a LAMA, and subsequently with a dual LABA/LAMA should symptoms worsen or exacerbation occur. Only if patients continue to suffer exacerbations do we suggest the addition of an ICS or a PDE-4 inhibitor, depending on patient profile/phenotype. It is possible that a subgroup of patients with COPD who have *raised blood or sputum eosinophils may respond better than others to ICS, although current data are still preliminary and somewhat contradictory.* Future studies are warranted to better define the groups who may benefit from ICS, and to identify the mechanisms by which bronchodilation reduces exacerbations.

## Abbreviations

A/F: Aclidinium/formoterol
b.i.d.: Twice daily
BDP/FF/GB: Beclomethasone dipropionate/formoterol fumarate/glycopyrronium bromide
CAMP: Cyclic adenosine monophosphate
COPD: Chronic obstructive pulmonary disease
EXACT: EXAcerbations of Chronic obstructive pulmonary disease Tool
FEV_1_: Forced expiratory volume in 1 second
FF/VI: Fluticasone furoate/vilanterol
GLY/F: Glycopyrronium/formoterol
GOLD: Global Initiative for Chronic Obstructive Lung Disease
HR: Hazard ratio
HRU: Healthcare resource utilization
ICS: Inhaled corticosteroids
IMPACT: InforMing the PAthway of COPD Treatment
IND/GLY: Indacaterol/Glycopyrronium
LABA: Long-acting β_2_-agonist
LAMA: Long-acting muscarinic antagonist
NAC: N-acteylcysteine
OR: Odds ratio
PDE-4: Phosphodiesterase-4
PR: Pulmonary rehabilitation
q.d.: Once daily
RR: Rate ratio
SCMC-Lys: Carbocysteine lysine salt monohydrate
SFC: Salmeterol/Fluticasone propionate combination
TIO/OLO: Tiotropium/Olodaterol
UMEC/VI: Umeclidinium/Vilanterol
